# Timing return-to-competition: a prospective registration of 45 different types of severe injuries in Germany’s highest football league

**DOI:** 10.1007/s00402-021-03854-8

**Published:** 2021-03-29

**Authors:** Werner Krutsch, Clemens Memmel, Volker Alt, Volker Krutsch, Tobias Tröß, Karen aus der Fünten, Tim Meyer

**Affiliations:** 1 Department of Trauma Surgery, University Medical Centre Regensburg, Franz-Josef-Strauss-Allee 11, 93053 Regensburg, Germany; 2 SportDocsFranken, Nuremberg, Germany; 3 Department of Pediatric Surgery and Pediatric Orthopedics, Clinic St. Hedwig Regensburg, Regensburg, Germany; 4 Department of Otorhinolaryngology, Paracelsus University Medical Centre Nuremberg, Nuremberg, Germany; 5 Institute for Sports and Preventive Medicine, Saarbrücken, Germany

**Keywords:** Severe injury, Professional, Soccer, Return-to-competition, Epidemiology, Media-based

## Abstract

**Introduction:**

Many professional football players sustain at least one severe injury over the course of their career. Because detailed epidemiological data on different severe injuries in professional football have been missing so far, this study describes the frequency and return-to-competition (RTC) periods of different types of severe football injuries.

**Material and methods:**

This epidemiological investigation is a prospective standardised injury analysis based on national media longitudinal registration. Injuries were classified according to the consensus statement by Fuller et al. (2006). The analysis includes injuries sustained by players of the first German football league during the seasons 2014–2015 to 2017–2018. Level of evidence: II.

**Results:**

Overall, 660 severe injuries were registered during the four seasons (mean 165 per season; 9.2 per season per team; incidence in 1000 h: 0.77). The body region most frequently affected by severe injury was the knee (30.0%; 49.5 injuries per season/SD 13.2) followed by the thigh (26.4%; 43.5 injuries/SD 4.2) and the ankle (16.7%; 27.5 injuries/SD 5.0). The distribution of injuries over the course of a season showed a trend for ACL ruptures to mainly occur at the beginning of a season (45.8%), overuse syndromes such as achillodynia (40.9%) and irritation of the knee (44.4%) during the winter months and severe muscle and ankle injuries at the end of a season. ACL ruptures showed the longest RTC durations (median 222 days).

**Conclusion:**

This study presents detailed epidemiological data on severe injuries in professional football. The body region most frequently affected by severe injuries was the knee. Several types of severe injuries showed a seasonal injury pattern. The appropriate timing of RTC after an injury is one of the most important and complex decisions to be made. This study provides information on the typical time loss due to specific severe football injuries, which may serve as a guideline.

## Introduction

Severe injuries are common in football and lead to considerable absence from training and competition [31]. They represent a serious problem for football players and clubs because of negative consequences such as absence from competition, potentially required surgical treatment or impaired physical performance after rehabilitation and re-integration. The epidemiology of some severe injuries in professional football, in particular ACL ruptures, has been well described in the literature [6, 20, 23]. Injury mechanisms and patterns as well as differences between age groups and genders have also been published for ACL injuries [5, 7, 16, 19, 20, 32] but less frequently for other types of severe injuries. Epidemiological information represents an important source for understanding the circumstances of an injury and for identifying the relevant risk factors and influencing factors [1, 26]. Epidemiological injury analyses in sports are generally aimed at furthering injury prevention strategies. Because of the negative consequences of severe football injuries their prevention has become highly important in professional football to reduce the days of absence and thus, to increase the availability of players—both essential factors for the success of a professional football team [8, 15].

Another important aspect of injury prevention in professional football is preventing re-injury after an index injury. Such re-injuries frequently occur after ACL injuries [31, 32] or muscle injuries [9, 30] and often result in a long absence from competition. The prevention of such re-injuries contains different targets during the rehabilitation phase, the re-integration of the player to training sessions as well as the decision about return-to-competition (RTC) in official matches [14]. Such decisions belong to secondary and tertiary prevention strategies and include the consideration of the most influential injury factors, mainly insufficient physical preparation [17, 18] and neuromotorical deficits due to a prematurely finished rehabilitation or early RTC after a previous injury [22]. In professional football, the decision on timing the RTC plays a crucial role because premature competition may result in re-injury or further complications. Criteria-based rehabilitation was recently published as an advancement compared to solely time-based rehabilitation [29] and both are used in the daily routine of current rehabilitation strategies after injuries. Detailed knowledge about the healing and rehabilitation phase of injuries, particularly of severe football injuries, is an essential part of injury management in professional football. The duration of absence and the timing of RTC for some specific injuries in professional football are well-known, especially in the case of ACL ruptures [24, 25, 28]. A recent study by Ekstrand et al. (2019) for the first time described a larger list of RTC timings for the 31 most frequent types of injury in the UEFA Champions League population [10]. However, severe injuries may be less frequent in “ordinary” professional football than in the UEFA Champions League. Severe injuries with longer time-out than 4 weeks in professional football player were less included in the above-mentioned study [10] and thus became the objective of the present study.

This study investigated all severe injuries in the German first football league that resulted in absence of more than 28 days [12]. The investigation was conducted by means of a national prospective injury registration in a standardised manner [19]. For the first time, the aim of this study was to investigate the frequency of severe injuries in national professional football and the subsequent length of absence from football. Among its objectives was to find out whether knee injuries represent the most frequent injury type in professional football and what the typical time loss is in these cases.

## Methods

This prospective cohort study (level of evidence: II) investigated severe football injuries in the first professional league in Germany (‘1st Bundesliga’) in longitudinal manner. The analysis only included injury types that resulted in absence from official football matches of at least 28 days and were therefore categorized as “severe” [12]. Data assessment only considered players with at least one official match during one of the seasons between 2014–2015 and 2017–2018. All injuries sustained in training sessions and official matches for clubs and national teams were included. Injuries were excluded if data definition by multiple media sources were not valid if absence from football matches were less than 28 days or injuries happened in players of a team, who played no single match during the season.

Injury data provided by medical staff and players are rarely available for several years as required for longitudinal studies. Therefore, injury data were prospectively documented in a standardised manner [19] by means of data obtained from the German kicker^®^ sports magazine that is published on a twice-weekly basis. Each professional football team has an assigned journalist who updates team-specific information. Additionally, we registered and analysed injuries by screening the social media websites (Facebook^®^ and Twitter^®^) of every first league team and players themselves as well as the online platform http://www.transfermarkt.de on a daily basis. Each registration of an injury in the database was followed by a verification process, and each information was confirmed by at least one other source. The diagnosis of registered injuries is best verified by medical staff according to the international guidelines [12, 13] as recently carried out by this study group [2, 3, 11, 20]. In the current study, the recently published standards for accurate injury registration by media analysis [19] were used to confirm the validity of media-based data (Table 1).

**Table 1:** Quality of media-based injury-specific and football-specific data of players with a severe injury ( adapted from Krutsch et al. 2019 [19])

Type of information	Valid media-based information	Not necessarily valid media-based information
Injury pattern	Injured body region (particularly of severe injuries)	Type of injury (particularly of minor injuries)
Anthropometric data	Age, weight and height	–
Football exposure	Match exposure	Training exposure (good estimates are possible)
Injury details	Match and training injury, time of injury and affected leg	Injury mechanism, contact or non-contact injury, foul and concomitant injuries
Football specific data	Dominant leg and position on field	Training and preparation data
Return-to-play issues	Time and level of RTC	Return-to-training, decision-making on RTC and RTC tests
Follow-up data after injuries	Recurrent time-out, playing level after 3-year or 5-years, time of career ending	Re-injuries, recurrent injuries, reduced performance and skills
Factors influencing injuries	Match frequency, change of coach or team and change of playing level	training frequency, training intensity and training contents

Injury types with the same diagnoses were collected and demonstrated in this study for affected body region, occurred injury type and seasonal distribution. For the illustration of the return to competition timing, selected this study the most frequent 45 specific injuries and injuries with a specified diagnosis and only these injuries were included in this question. In addition to injury patterns, the current study also investigated the distribution of specific football injuries over the course of a season. Injury incidence was measured in 1000 football hours (h) of training, match and overall exposure (training and match injuries combined). Official sports media provide valid information about the match exposure of every football player over the course of a season. However, this is not the case for training exposure that is why it was calculated according to previous publications by the authors wherein the same study population within a different study period (seasons 2008–2010) was used and to own experience due to playing at professional football levels [2, 19]. Based on this, training exposure was calculated to average 7200 h per team per year and 340 h per player a year. The average match exposure amounted to 50 h per season. RTC timings were calculated from the day of injury to playing the first official match-information that is reliably available from public media.

Because the entire data pool was exclusively derived from publicly available media data on injuries and players, this study did not have to be approved by the local Ethics Committee. Continuous data are expressed as means ± standard deviations (SD) and categorical data as frequency counts (percentages). Injury classification for tables was inspired by OSICS/Orchard sports injury classification system.. All analyses were carried out with IBM SPSS Statistics, version 24.0 and R (version 3.3.3, The R Foundation for Statistical Computing).

## Results

**Fig. 1:**
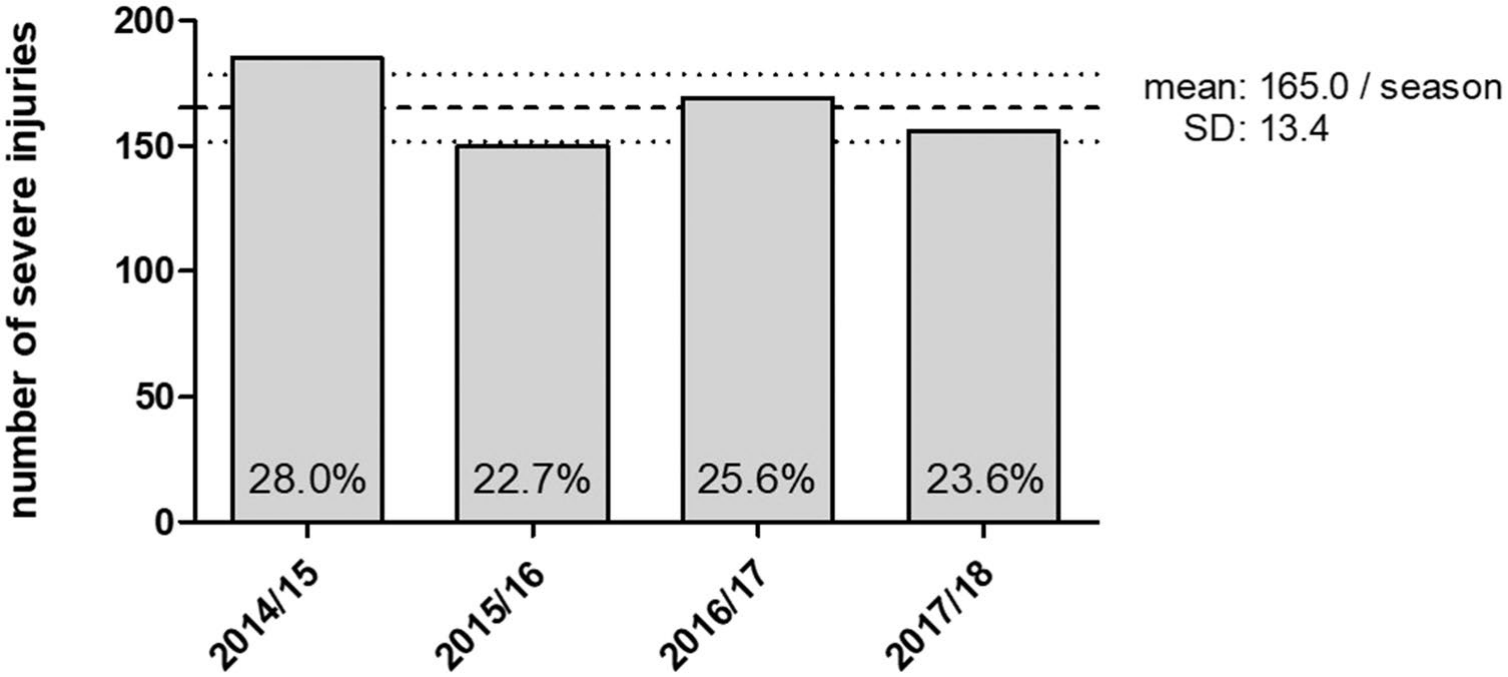
Number (percentage) of severe injuries during the four seasons; dashed lines marks mean injury frequency ± standard deviation

Overall, 660 severe injuries occurred during the study period of four ‘Bundesliga’ seasons (mean 165 per season or 9.2 per season per team, injury incidence in 1000 h football exposure 0.77). The frequency of severe injuries among all injuries varied over the study period between 22.7 and 28.0% (Fig. 1). The mean age of the injured players was 26.3 years (SD 4.0) Injury distribution according to playing position is displayed in Table 2.

**Table 2:** Anthropometric data of the players

Anthropometric data	Mean	SD	Min	Max
Age at injury (year)	26.3	4.0	17.6	38.6
Height (cm)	183.0	6.7	165	198
Weight (kg)	78.5	6.9	61	98
Position on field	*n*	%		
Keeper	26	4.7		
Defender	174	31.6		
Midfielder	242	43.9		
Striker	109	19.8		

The body region most frequently affected by severe injuries was the knee joint (30.0%; 49.5 injuries per season/SD 13.2) followed by the thigh (26.4%; 43.5 injuries per season/SD 4.2), the ankle region (16.7%; 27.5 injuries per season/SD 5.0) and the lower leg (6.2%; 10.3 injuries per season/SD 2.8). 5 severe head traumas like concussion with combined midfacial fractures (5 cases) also led to severe injuries (Table 3). The most frequent specific severe injuries in professional football were muscle tears (19.8%) and tendons rupture of the thigh (5.3%), lateral ligament injuries of the ankle (6.5%) and medial collateral ligament injuries of the knee joint (5.1%). Only two out of the 16 most common severe injury types did not affect the lower limbs, one was located at the lower back (low back pain) and one at the acromioclavicular joint (dislocation: 2.4% each) (Table 4).

**Table 3:** Severe injuries—most frequently affected body regions

Body region	*n* (660)	*n* per season	% of total (660)
Knee	198	49.5	30.0
Thigh	174	43.5	26.4
Ankle/achilles tendon	110	27.5	16.7
Lower leg	41	10.3	6.2
Foot/toe	36	9.0	5.5
Shoulder/clavicle	27	6.8	4.1
Lower back/pelvis/sacrum	20	5.0	3.0
Hip/groin	20	5.0	3.0
Sternum/ribs/upper back	9	2.3	1.4
Elbow	7	1.8	1.1
Head/face	5	1.3	0.8
Hand/finger/thumb	5	1.3	0.8
Abdomen	5	1.3	0.8
Neck/cervical spine	2	0.5	0.3
Forearm	1	0.3	0.2

**Table 4:** The 16 most common specific types of severe injuries in professional football

Specific type of injury	*n* (432)	*n* per season	% of total (551)
Muscle tear, thigh	109	27.3	19.8
Lat ligament(s) rupture, ankle	36	9.0	6.5
Tendon rupture, thigh	29	7.3	5.3
MCL rupture, knee	28	7.0	5.1
Meniscal tear	27	6.8	4.9
Muscle pain, thigh	26	6.5	4.7
ACL rupture	24	6.0	4.4
Syndesmosis rupture, ankle	23	5.8	4.2
Achillodynia	22	5.5	4.0
Irritation, knee	18	4.5	3.3
Fracture, foot	17	4.3	3.1
LCL rupture, knee	16	4.0	2.9
Muscle tear, lower leg	16	4.0	2.9
Cartilage damage, knee	15	3.8	2.7
Lower back pain	13	3.3	2.4
AC joint injury	13	3.3	2.4

*MCL* medical collateral ligament, *LCL* lateral collateral ligament, *AC*, acromioclavicular

**Fig. 2:**
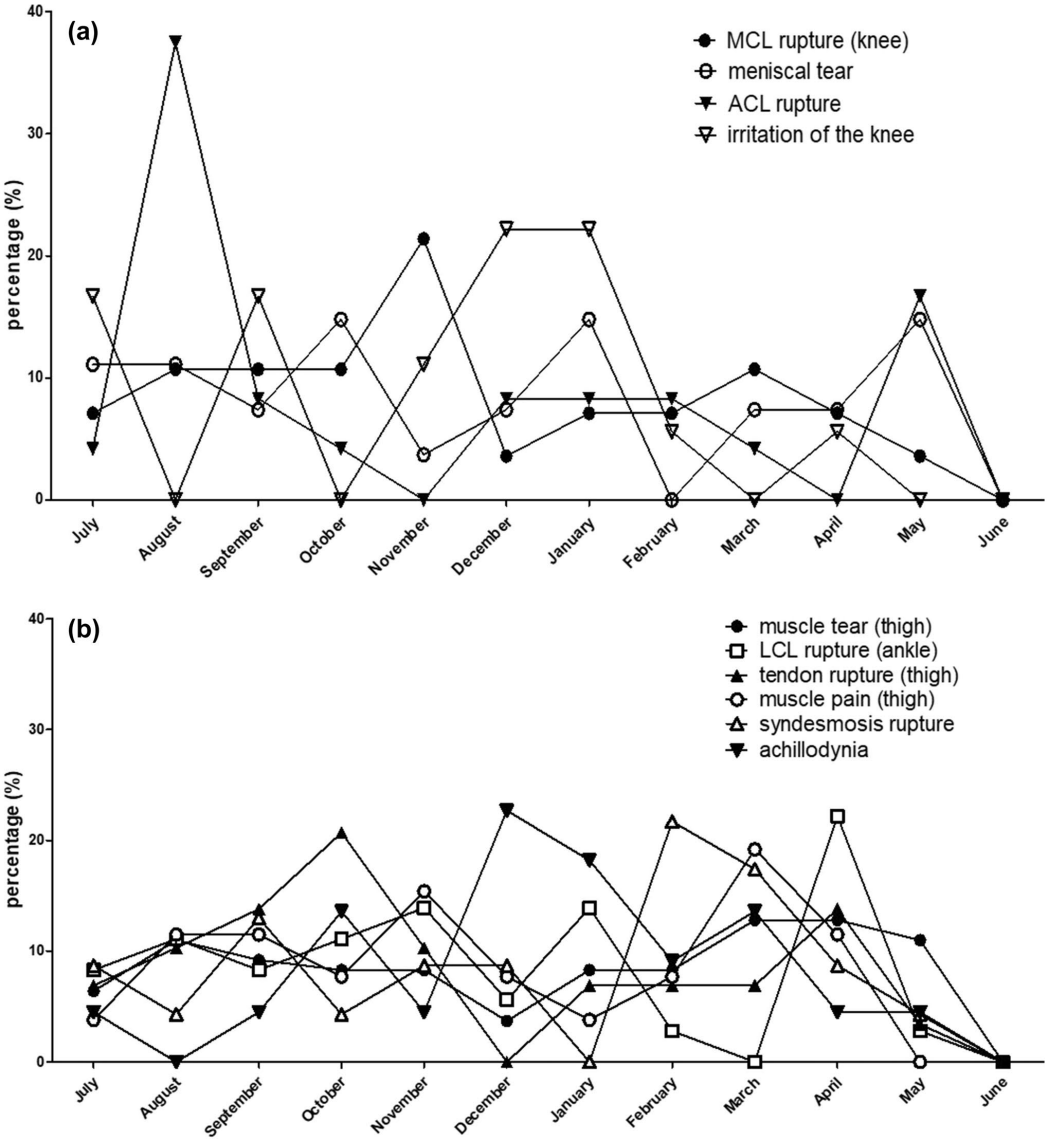
**a** Seasonal distribution of the most common severe injuries of the knee. **b** Seasonal distribution of the most common severe injuries of thigh and ankle region

Seasonal distribution showed a trend towards an increasing frequency of muscles tears at the end of the season (March–May 36.6%). Similar results were found for thigh muscle pain (March–April 30.7%) and lateral ankle ligament ruptures (April 22.2%). The frequency of ACL injuries was higher at the beginning of the seasons (Aug–Sept 45.8%). Overuse injuries such as achillodynia (Dec–Jan 40.9%) or ‘irritation of the knee’ (Nov–Jan 55.5%) mainly occurred during the winter months (Fig. 2a, b).

For the calculation of RTC timings in specific diagnoses, 109 injuries were excluded as no specific diagnosis could be established. The remaining 551 injuries (137.8 injuries per season/SD 12.7) could be grouped in 45 specific injury diagnoses. Of the 45 types of specific injury diagnosis for the RTC calculation, 12 affected the knee joint and 7 the ankle*.* The injury type with the longest median absence of all severe injuries was an ACL rupture, it lasted 222 days. Other knee injuries involving absence from football of more than three months were cartilage injury of the knee joint, a PCL (posterior cruciate ligament) rupture and a rupture of the patellar tendon. Further injuries with long absence were a cartilage damage of the hip joint, a disc prolapse, a rupture of the Achilles tendon, a medial ligament injury of the ankle and fractures of the ankle or fibula shaft. Other fractures such as face, upper extremities, ribs or clavicle fractures showed shorter RTC times of less than 2 months (Table 5).

**Table 5:** Severe injuries arranged by the length of the return-to-competition period

Severe injury types	*n*	Mean (d)	SD (d)	Median (d)	Min (d)	Max (d)
ACL rupture	24	216.3	43.8	221.5	124	284
Cartilage damage, hip	4	213.3	67.4	181.5	161	329
Disc prolapse	2	210.5	56.5	210.5	154	267
Achilles tendon rupture	4	184.0	48.6	205.0	101	225
Cartilage damage, knee	15	141.5	153.1	88.0	29	629
MCL rupture, ankle	2	125.5	30.5	125.5	95	156
Osteitis pubis	10	114.2	90.4	70.0	40	327
Patellar tendon rupture	1	111.0	0.0	111.0	111	111
Ankle fracture	6	108.8	73.7	95.0	34	258
Fibular shaft fracture	3	104.3	65.7	63.0	53	197
PCL rupture	5	101.8	61.3	84.0	34	208
ACL partial rupture	7	98.9	57.4	81.0	41	224
Tendon rupture, thigh	29	86.0	32.0	83.0	32	157
Abdominal muscle strain	3	79.7	30.1	74.0	46	119
Tibial fracture	3	75.7	35.4	69.0	36	122
Irritation knee	18	75.2	33.0	68.0	31	124
Foot fracture	17	75.1	50.3	63.0	31	238
Lower back pain	13	74.2	55.5	54.0	30	227
Meniscal tear	27	73.7	60.2	51.0	31	327
Tendinopathy patellae	9	72.0	27.2	75.0	31	108
Syndesmosis rupture	23	71.2	25.7	63.0	36	118
Shoulder dislocation	6	69.2	10.2	65.5	58	83
Ligament rupture, elbow	3	66.7	7.5	72.0	56	72
Cartilage damage, ankle	3	66.3	31.3	61.0	31	107
LCL rupture, knee	16	64.2	21.3	69.5	32	95
MCL rupture, knee	28	58.9	27.0	51.5	30	155
Facial fracture	5	58.0	17.5	57.0	28	80
Achillodynia	22	56.7	36.0	40.0	30	176
Hamstring strain	4	55.5	16.6	55.5	34	77
MCL strain knee	7	54.4	30.9	43.0	28	118
Contusion foot or toe	7	53.7	19.9	65.0	30	83
Lat ligament(s) rupture, ankle	36	52.9	29.9	41.5	28	165
Hand fracture	5	51.4	13.9	50.0	30	73
Muscle tear, thigh	109	50.0	31.3	41.0	25	236
Muscle pain thigh	26	48.7	21.3	40.0	28	114
AC joint injury	13	44.8	16.2	41.0	29	86
Clavicle fracture	5	43.2	8.7	46.0	32	53
Rib fracture	6	43.2	19.7	35.5	30	87
LCL strain knee	2	43.0	12.0	43.0	31	55
Inguinal hernia	1	43.0	0.0	43.0	43	43
Muscle tear, calf	16	42.9	12.5	38.0	30	67
Muscle strain, groin	2	41.0	10.0	41.0	31	51
Forearm fracture	1	41.0	0.0	41.0	41	41
Muscle tear, abdomen	2	36.5	2.5	36.5	34	39
Spine fracture	1	31.0	0.0	31.0	31	31
Total	**551**					

## Discussion

For the first time, this study provides longitudinal prospective data on severe injuries in national professional football. Epidemiological injury reports so far have only covered a few types of injury, such as ACL injury [19, 20, 31] or concussion [3, 4, 27]. This study describes the injury incidence, the injury frequency over the course of a season and the RTC times of several severe injuries in professional football in detail.

### Frequency of severe injuries in professional football

The frequency of specific types of severe injury in this cohort resembles those found in previous studies, for instance for ACL ruptures [19, 31], fractures [21] and muscle injuries [9]. A new and important addition to the current knowledge is the finding that normally less severe injuries such as concussions with a mean absence from professional football of just a few days [3, 4, 10] and a recommended period for a safe return-to-play of 7 days can also result in long absences from football when combined with midfacial fractures. Some injury types seem to peak at a specific time of the season. Such knowledge is important to better understand the influencing factors for the occurrence of injuries as a basis for well-balanced and well-timed prevention strategies. It has been shown that ACL injuries seem to require prevention strategies before the beginning of a season as they peak at that time [20]. The current study presents novel knowledge on the distribution of other types of severe injuries over the course of a season, too. The steady increase in the number of severe muscle and ankle injuries towards the end of a season could be a consequence of a football season’s cumulated stressors and, thus, a result of fatigue and overstress of the muscles and reduced neuromotor adaptation of the joints. The increased frequency of overuse injuries in the winter months raises the suspicion that temperature changes and pitch conditions may have an impact.

### Return-to-football after a severe injury

So far, only Ekstrand et al. (2019) have provided a detailed list of RTC times of different football injuries [10]. The mean RTC timings in the UEFA European football level seems to show bit faster RTC timings, e.g. in case of ACL injuries, compared to national professional football in this study. This study gives RTC data for 45 different specific types of injury in professional football representing the largest number of RTC times that has been published so far. Such publications are important: despite the novel development of criteria-based rehabilitation after injury, which includes repeated testing of different parameters and specific criteria on RTC [29], team doctors, sports physicians and sports physiotherapists in football benefit from knowing the time range between minimum and maximum RTC times for individual injuries, particularly in the case of less frequent (and therefore less familiar) severe ones. Such knowledge represents valuable information also for non-medicals like the player as well as for the team (coach) and the club [22]. Additionally, the RTC times of different injuries in such cohort of professional football may also be used as a minimum benchmark for the RTC time after specific injuries of amateur football players. It seems to be unrealistic to expect amateur footballers to benefit from the same RTC timings after injury as professionals. Amateur football has shown in previous studies higher re-injury rates compared to professional football [14], which underlines the potential benefit of guiding values for RTC at these levels.

### Perspectives for injury prevention

Preventing severe injuries is essential for further development of modern football with increasing professionalism worldwide [20], for reducing numbers and duration of injuries and their potentially subsequent long-term health sequelae and for lowering the dropout rate of injured player and health costs [8, 15]. Knowledge about frequency and potential influencing factors is the basis for developing suitable prevention strategies [1]. This study not only provides advanced knowledge about factors influencing severe injuries as part of primary prevention but contains also helpful information on RTC times as part of secondary prevention. According to this study, knee injuries often have to be classified as severe as they frequently cause a time loss of more than 4 weeks. This does not solely refer to traumatic injuries but to overuse injuries alike examples being ‘irritations of the knee’ or an isolated meniscal injury. For a prevention strategy, the timing of injuries during the season is valuable to know, as some injuries occur mainly at the beginning of the season (e.g. ACL), some during the winter months (e.g. irritation).

### Methodology and limitations

Previous media-based studies often had some limitations with regard to the definition of injuries as well as the inclusion and exclusion criteria, which may have led to imprecise injury statistics and debatable conclusions. The current study population was evaluated by means of an advanced national, regional and local media analysis with strict exclusion criteria and validation process (Table 1) [19, ]. As this media-based analysis was prospectively conducted over 4 years, which simplified the injury recording process as the most detailed information can be obtained immediately after the occurrence of an injury. Therefore, the strengths of this study are the verification of the injury diagnosis by different public sources, its precisely defined methodology and its differentiation between valid and weak information on professional football players. Injury and RTC times of severe injuries can be validly documented via media sources. Nevertheless, the gold standard of injury registration in football remains injury reports provided by the clubs’ medical staff, (which is also not free from limitations itself of course). Not just ACL injuries [19] also other severe injuries showed high external validity in the verification process. Only a minority (16.5%) of registered media-based injuries had to be excluded for the calculation of RTC timings. Furthermore, media analysis allows for a very good estimate of the match injury incidence analysis and a good estimation of the training exposure as shown in earlier publications in the same study population [2, 3, 19]. Media-based data should be critically enquired and validated against various media sources for each single injury.

## Conclusion

For the first time, this study presents epidemiological data of severe injuries of professional football players. Severe injuries with a long period of time-out in football mainly affected the knee. There is a seasonal accumulation of ACL injuries at the beginning of the season. The appropriate timing of return-to-competition after an injury is one of the most important and complex decisions to be made by the medical team. Knowledge about the typical time loss may serve as a guideline, particularly in the case of uncommon injuries or less experienced medical staff.
